# Analysing disability policy in Namibia: An occupational justice perspective

**DOI:** 10.4102/ajod.v7i0.401

**Published:** 2018-07-31

**Authors:** Tongai F. Chichaya, Robin W.E. Joubert, Mary Ann McColl

**Affiliations:** 1 Department of Occupational Therapy, School of Health Science, University of KwaZulu-Natal, South Africa; 2 Canadian Disability Policy Alliance, Canada; 3 Centre for Health Services and Policy Research; Rehabilitation Therapy/Public Health Sciences, Queen’s University, Canada

## Abstract

**Background:**

The Namibian disability policy of 1997 has not been reviewed for about 20 years, which has raised concerns with persons with disabilities and stakeholders in the fields of disability and rehabilitation. In March 2017, the government publicised its intention to review the policy. Thus, this study’s purpose was to generate evidence that can contribute to the development of a more current disability policy that will promote occupational justice.

**Objectives:**

The aim of the study was to develop an alternative disability policy option for Namibia and to present outcomes and trade-offs using a policy analysis approach while applying the occupational justice framework to gather evidence.

**Method:**

A qualitative research design and Bardach’s eightfold path approach to policy analysis were used. Critical disability theory provided the theoretical framework. The occupational justice framework was the conceptual framework for the study. Evidence from preceding phases of this study and appropriate literature was utilised to construct possible disability policy alternatives in Namibia, set evaluative criteria, project outcomes and confront trade-offs.

**Results:**

Three main disability policy alternatives emerged: access policy, support policy and universal coverage policy. Access policy had the fewest trade-offs, and the support policy had the most trade-offs in the Namibian context. Access policy was projected to foster occupational participation among persons with disabilities.

**Conclusion:**

Results have implications for selecting disability policy alternatives that promote occupational participation and justice among persons with disabilities in Namibia. Furthermore, the study has implications for advancing the practice of occupational justice in disability policy formulation.

## Introduction

The national policy on disability in the Government of the Republic of Namibia (GRN 1997) has not been reviewed since its inception in 1997. However, there have been several changes in the disability field, both locally and internationally, to which the current policy has not been responsive. Typically, policies are reviewed after every 5 years, which means that the national policy on disability may not be responsive to the current needs and rights of persons with disability. According to the national Namibia Population and Housing Census of 2011, there were 98 413 persons with disabilities across the 14 administrative regions of the country, accounting for approximately 5.0% of the total population (GRN 2012). However, these statistics are questionable because the census questionnaire was impairment based and many disabilities such as those linked to developmental and mental conditions remain unnoticed or unreported. The increasing prevalence of non-communicable diseases, high percentage of road traffic accidents, high prevalence of HIV and TB as well as a poverty incidence of 26.9% (GRN 2015) suggest that the actual percentages of persons with disabilities are higher than the global estimations of 15.0% of the population.

Persons with disabilities in Namibia have expressed dissatisfaction and frustration with their current life experiences (GRN 2008; Haidula 2016; Sankwasa 2015; Sibeene 2008). The undesirable life aspects experienced by persons with disabilities in Namibia can be traced to local disability policy shortfalls. Stakeholders in disability and rehabilitation services agree that there is a need to review the disability policy; the government recently publicised its intentions to do this (GRN 2017a). The purpose of this study was to generate timely evidence on possible disability policy alternatives that could be used in disability policy formulation in Namibia by applying the occupational justice framework to a step-by-step policy analysis approach initially proposed by Bardach (2000), namely the eightfold path to more effective problem-solving.

## Objectives of the study

The aim of the study was to develop alternative disability policy options for Namibia and to present outcomes and trade-offs using a policy analysis approach while applying the occupational justice framework to gather and critique evidence.

## Contextual and theoretical background to the study

The human rights model of disability, Namibia’s disability policy environment, an occupational justice framework, critical disability theory and the eightfold path to disability policy analysis are presented in this section. The human rights model of disability is used in this study to support the theoretical and conceptual frameworks.

## Human rights model of disability

Disability is an evolving, complex concept. Defining it is complicated and controversial, often taking the dimension of a particular model or purpose for which it is being used. Over the years many disability models have been used. These include the medical model, the charity model, the economic model, the social model, the bio-psychosocial model and more recently the human rights model (Hughes & Paterson 1997; Mpofu & Oakland 2010; Shakespeare & Watson 1997).

The human rights model, which is currently emerging in Africa, considers disability to be a human rights issue, based on the notion that all human beings are equal and have rights that must be respected (Vanhala 2011). This model is the first to use moral principles as a basis for disability policy (Degener 2014). People with disabilities are citizens and as such have the same rights as any other citizen. All actions to support persons with disabilities should therefore be rights based. This approach has merit the world over and more so in Africa, where persons with disabilities have been subjected to extreme inequality. Namibia signed the United Nations Convention on the Rights of Persons with Disabilities (UNCRPD) and its optional protocol; therefore, a rights approach provides a basis for disability policy formulation in Namibia.

This study adopted the perspective of the human rights model of disability, because it is based on moral principles and values as the base for disability policy. Similarly, occupational justice emphasises that every individual has a right to participate in occupations of their choice and need. Levelling the ‘playing field’ is underpinned in the human rights-based approach to disability so that persons with disabilities can access and participate in the livelihood, education, socio-economic, cultural, political and health sectors as equal citizens. In essence, this model caters for both civil and political rights, as well as economic and cultural rights. This includes removing physical and social barriers and bringing about attitude adjustment among policymakers, service providers and family members, with the aim to have a society in which all persons with disabilities have the freedom and necessary resources to participate in occupations of their need and choice.

## Disability policy environment in Namibia

Since Namibia’s political independence in 1994, the government has enacted initiatives with the aim of improving the lives of persons with disabilities. These include introduction of a national disability policy in 1997, establishment of a Disability Unit in the Office of the Prime Minister in 2001, and passing an act for the establishment of the National Disability Council in 2004 (members of the council were later appointed in 2012). Transfer of disability services from the Ministry of Lands, Resettlement and Rehabilitation to the Ministry of Health and Social Services in 2005 resulted in two rehabilitation divisions within the ministry, one division under primary healthcare and the other under social services. In 2015, a new Department of Disability Affairs was formed in the Office of the Vice President. Three entities – the Rehabilitation Division under social services, the Disability Unit from the Office of the Prime Minister and the National Disability Council – were transferred to this new department. The aim of the move to bring the three entities under one department was to streamline functions that relate to the empowerment of persons with disabilities and to correct the duplication of efforts that was happening in the Ministry of Health and Social Services. Furthermore, the move aimed to reverse antagonism among personnel in the three entities, which were perceived to be causing complaints among persons with disabilities over the past years. The impact of this move on the lives of persons with disabilities is yet to be seen. The historical juggling of the disability services between various (and sometimes unsuitable) government departments, as described above, is also indicative of gaps in the local disability policy.

The current disability policy was developed by a European freelance consultant who had a 2-year exposure in Namibia. The suitability of such a consultant in developing a responsive local disability policy is debatable. Typically, policy development is a systematic process that is based on evidence and wide consultation with stakeholders involved (Buse 2008). There is no evidence of a systematic process that was followed when the policy was developed. Furthermore, there is no evidence of involvement of the disability movement in the policy formulation. The disability policy was developed when disability services were under the Ministry of Lands, Resettlement and Rehabilitation. This ministry no longer exists, which contributes to lack of ownership of the policy among stakeholders.

The disability policy preceded publication of the UNCRPD and the occupational justice framework; thus, the policy does not fully address the rights for occupational participation among persons with disabilities outlined in the latter. In March 2017, the Namibian government publicised its intentions to review the disability policy (GRN 2017a). In view of this it was considered important to conduct research that may contribute to the local evidence essential for disability policy formulation.

## Occupational justice framework

Occupational justice is an evolving concept that focuses on the fair, civic, political and moral empowerment of people to participate in occupations, broadly defined as all human doings in context, that they need to live, to choose and find meaning in life. Therefore, participation in occupations is a rights issue; every person has a right to participate in occupations that are meaningful and can positively contribute to their well-being and to society at large (Hocking 2017). When circumstances external to a person hinder occupational participation the result is occupational injustice. The occupational justice framework was developed to provide an outline of the forces that interplay, leading to outcomes of either occupational justice or occupational injustice (Townsend & Polatajko 2013; Wilcock & Townsend 2000; 2009). Components of the occupational justice framework and the participatory occupational justice framework include structural and contextual factors (Stadnyk, Townsend & Wilcock 2010; Whiteford & Townsend 2011). Structural factors are divided into underlying occupational determinants such as international or national policies and occupational instruments that enable participation in necessary or desirable occupations such as education, employment and technology. Examples of contextual factors are national origin, ethnicity and disability. The occupational justice framework can be applied with individuals, groups, and communities (Townsend & Marval 2013). To date there is no literature on the applications of this framework in disability policy formulation. Such an application has potential because policy forms part of underlying occupational determinants, and policies create an environment for promoting occupational justice or occupational injustice.

## Critical disability theory

Critical disability theory was selected as the overarching theoretical framework to explain how circumstances may empower or enslave persons with disabilities. For this study, critical disability theory in particular was selected from a diverse family of critical theories derived from the work of Max Horkheimer (Horkheimer 1982; Hosking 2008). Disability is a complex social construct that requires the social environment to be addressed beyond a person’s impairment. Critical disability theory describes persons with disabilities as traditionally ‘oppressed’; this is because society treats them in ways that diminish their social, personal, physical, and financial well-being, and they are viewed as members of a socially disadvantaged minority group (Charlton 1998; Devlin & Pothier 2005). Suppression and marginalisation of persons with disabilities cannot be as dogmatically accepted as they appear to be in society. A critical disability theory lens provides a guide to examining and redressing oppressive social factors that tend to be unconsciously accepted.

## Applicability of the eightfold path to disability policy analysis

**FIGURE 1:**
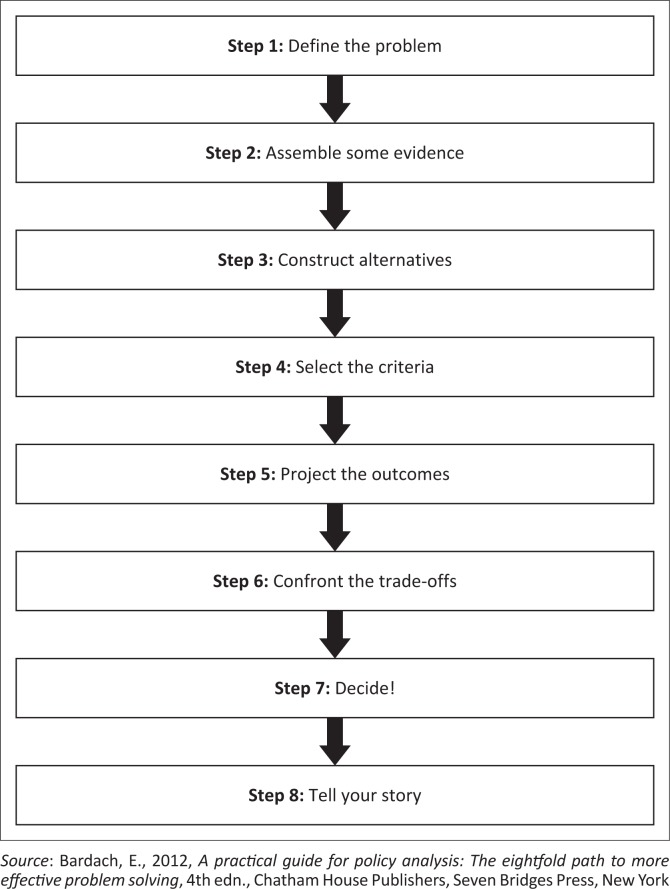
Eightfold path for policy analysis.

Policy analysis is complex and time-consuming; thus, for this study, the eightfold path for policy analysis was used because it allows for a more structured approach to conduct policy analysis effectively (Bardach 2012). The eightfold path for policy analysis has eight steps. Step 1 defines the problem; Step 2 involves assembling evidence; Step 3 covers constructing alternatives; Step 4 entails selecting criteria; Step 5 involves projecting the outcomes; Step 6 pertains to confronting trade-offs; Step 7 entails decisions; and Step 8 involves telling one’s story. The literature reveals that Bardach’s approach to policy analysis has been used in social policies, income inequality–addressing policies, public health policy, energy sector policies and agricultural policies (Bardach 2012; Kanna 2006; Weiner 2008). However, no study has been identified in which this approach was used in disability policy. The presence of complex policy-related barriers to occupational participation faced by persons with disabilities thus presents an opportunity for using this policy analysis approach. Figure 1 shows the steps of the policy analysis approach.

## Research method and design

This study was completed in two phases, both of which used a qualitative analytical study design. Both phases used the policy analysis approach suggested by Bardach (2000). The Bardach approach uses a policy analysis strategy that seeks to generate recommendations for policy alternatives based on gathered evidence. The occupational justice framework provided the conceptual framework, and critical disability theory guided the underlying reasoning behind the study. Using an outcomes matrix, proposed disability policy alternatives were presented against the set evaluative criteria. Trade-offs for each policy alternative were presented using scores for each criterion. The evidence used in this study was based on the findings of two systematic studies that preceded this study, as well as literature findings (Chichaya, Joubert & McColl 2017a; 2017b).

## Ethical consideration

Ethics clearance to conduct the study was obtained from the Humanities and Social Science Research Ethics Committee of the University of KwaZulu-Natal (HSS/0078/015D) and the Ministry of Health and Social Services Research Management Committee in Namibia. Principles of respect for persons and beneficence and justice were upheld throughout the study.

## Trustworthiness

Trustworthiness was ensured by carefully using two frameworks and an evidence matrix to assess the current policy and propose policy alternatives. Data triangulation was achieved through the use of literature and three participant groups who provided responses on disability policy in Namibia. In view of their experience conformability was addressed by two supervisory collaborators. They validated, reviewed and provided additional input in terms of what the first author had written. To enable transferability of findings to other settings, the study provides a description of the Namibian disability policy environment. Transferability is dependent on the context being considered. For example, transferability of the findings to other Southern African countries with similar socio-economic, political and cultural backgrounds is more likely as opposed to Western countries.

### Phase 1: Findings from the first study

Phase 1 used Steps 1 and 2 of the eightfold path for policy analysis. Step 1 focused on defining the disability policy problems and Step 2 was for gathering evidence from the literature and the participants in Namibia. The first study was a desk-based analysis of the Namibian disability policy and a comparative analysis of disability policies of other Southern African nations using the UNCRPD as a yardstick. Findings from the first study revealed occupational injustice embedded in the discourse of the Namibian disability policy. Four forms of occupational injustice were identified in the study. Occupational marginalisation was present because of non-existence of evidence of active involvement of persons with disabilities in the formulation of the current disability policy in Namibia. Occupational deprivation persists as evidenced by the absence of state influence in ensuring accessible transport services for persons with disabilities. The latter are predominantly privately owned, and this results in the wide prevalence of inaccessible transport for persons with disabilities, even for those who can pay for transport. This significantly contributes to their isolation and occupational deprivation because they are denied opportunities to reach schools, workplaces and marketplaces for occupational participation. Occupational alienation is a form of injustice that refers to a sense of isolation and absence of meaning in occupational participation. Persons with hearing impairment, who for many years could only reach Grade 10 and have had to settle for menial tasks, are at risk of occupational alienation because of an absence of Grade 12 and tertiary education for learners with hearing impairments, which could lead to professional qualifications and better jobs. This can be traced to the disability policy not adequately addressing the educational needs of persons with hearing impairment.

Occupational inconsideration will be introduced more fully in the discussion to interpret some elements of the study findings. This concept will be proposed as a newly named form of occupational injustice. Occupational inconsideration refers to injustice whereby those in authority or in disability policy decision-making positions knowingly or unknowingly disregard the centrality of occupation in formulating disability policies.

Furthermore, the policy predominantly contains the passive voice. This limits implementation because when the passive voice is used there is no identification of who should take the responsibility to act. The frequent use of the word ‘shall’ in the disability policy makes it ambiguous. This implicitly propagates a background for the proliferation of occupational injustice faced by persons with disabilities in Namibia.

Namibia has not yet submitted the initial UNCRPD report, which was due in 2010 (UN 2018). This delay in submission of reports to some extent indicates lack of prioritisation to demonstrate domestication of the UNCRPD. Late submission is not unique to Namibia but includes other Southern African countries. Lack of expertise and resources have been cited as reasons for slow advancement on disability policies (UN 2018).

### Phase 2: Description of participants for Phase 2

Phase 2 of the study continued Step 2 of the Bardach approach (2012), focusing on gathering evidence. The second study provided evidence gathered through interviews with 15 persons with disabilities, 8 disability policy decision-makers, as well as 2 focus groups with a combined total of 17 occupational therapists who had an interest in and understanding of occupational justice. Tables 1 and 2 describe the 15 persons with disabilities and decision-makers who participated in the study; Table 3 describes the 17 occupational therapists.

**TABLE 1:** Summarised description of the 15 persons with disabilities interviewed.

Participant ID	Gender	Age	Impairment type	Onset (years)	Urban/rural	Occupation
CIT1	M	50	Paraplegia	20	Rural	Unemployed
CIT2	M	35	Congenital malformations	35	Rural	Unemployed
CIT3	M	21	Paraplegia	3	Urban	University student
CIT4	M	49	Tetraplegia	24	Urban	Unemployed
CIT5	M	38	Post-polio syndrome	34	Urban	Business analyst
CIT6	F	28	Post-polio syndrome	26	Urban	Office administrator
CIT7	F	35	Cerebral palsy	35	Urban	Communications officer
CIT8	F	45	Chronic schizophrenia	18	Urban	Unemployed
CIT9	F	24	Congenital malformations	24	Urban	University student
CIT10	M	18	Hearing impairment	16	Urban	Unemployed
CIT11	F	31	Visual impairment	31	Rural	Unemployed
CIT12	M	19	Bilateral L/L amputations	6	Rural	Unemployed
CIT13	F	55	Hemiplegia	3	Rural	Unemployed
CIT14	M	38	Learning disabilities	38	Rural	Unemployed
CIT15	F	33	Traumatic brain injury	8	Rural	Domestic worker

*Source*: Chichaya, T.F., Joubert, R.W. & McColl, M.A., 2017b, *Voices on disability in Namibia: Evidence for entrenching occupational justice in disability policy formulation*, Manuscript submitted for publication

CIT, Citizen referring to the persons with disabilities who participated in the study; M, male; F, female.

**TABLE 2:** Summarised description of eight key informants (decision-makers).

Participant ID	Gender	Number of years in disability/rehabilitation services	Organisation/position at time of interview
KI:1	M	11	National coordinator
KI:2	F	26	Rehabilitation service manager
KI:3	M	25	Advisor on disability issues
KI:4	F	12	National Disability Council
KI:5	F	20	Office of Vice President – Disability Affairs
KI:6	F	24	National manager
KI:7	M	6	Organisation for Persons with Disabilities leader
KI:8	F	8	Organisation for Persons with Disabilities leader

*Source*: Chichaya, T.F., Joubert, R.W. & McColl, M.A., 2017b, *Voices on disability in Namibia: Evidence for entrenching occupational justice in disability policy formulation,* Manuscript submitted for publication

KI, key Informant meaning the disability policy decision makers; M, male; F, female.

**TABLE 3:** Summarised description of 17 occupational therapists.

Participant ID	Gender	Years of experience	Setting	Public sector experience	Private sector experience
**Focus group 1 participants**					
1FG1	F	10	General OT, Community OT and Hands Therapy	X	-
1FG2	F	2	Rotational	X	-
1FG3	F	3	Forensic Psychiatry, Paediatrics and Orthopaedics	X	X
1FG4	F	3	General OT and Hands therapy	-	X
1FG5	F	9	Civil Psychiatry	X	-
1FG6	F	9	Paediatrics, Wheelchairs, NGOs	X	X
1FG7	M	11	General OT and Forensic Psychiatry	X	-
1FG8	F	4	Learning Disabilities and General OT	X	X
1FG9	M	4	General OT and Psychiatry	X	-
**Focus group 2 participants**					
2FG1	F	5	Spinal Cord Injury Rehabilitation	X	-
2FG2	F	10	Social Services and General Rehabilitation	X	-
2FG3	F	2	Rotational	X	-
2FG4	F	3	General OT and Paediatrics	X	X
2FG5	F	12	Physical Rehabilitation	X	X
2FG6	M	3	Work Rehabilitation and Psychiatry	X	X
2FG7	F	3	Paediatrics and School	X	X
2FG8	F	4	General OT	-	X

*Source*: Chichaya, T.F., Joubert, R.W. & McColl, M.A., 2017b, *Voices on disability in Namibia: Evidence for entrenching occupational justice in disability policy formulation*, Manuscript submitted for publication

1FG, First focus group; 2FG, Second focus group; M, male; F, female.

### Phase 2: Findings from the second study

Barriers to occupational participation viewed as occupational injustice experienced by persons with disabilities in Namibia were divided into five categories: physical barriers; access barriers; expertise barriers; systemic barriers; and attitudinal barriers. There was a discrepancy of perceptions between disability policy decision-makers and persons with disabilities on occupational participation barriers experienced by persons with disabilities in Namibia, as well as their suggested policy changes. Persons with disabilities expressed more hindrances to occupational participation than those mentioned by disability policy decision-makers. There was far greater concurrence between perceptions of barriers among people with disabilities and occupational therapists. The discourse among persons with disabilities revealed a life of struggle and disadvantage. Table 4 shows the barriers to occupational participation mentioned by each participant group.

**TABLE 4:** Barriers to occupational participation mentioned by each participant group.

Barriers to occupational participation mentioned among participant groups	Persons with disabilities	Decision-makers	Occupational therapists
Barriers to accessing assistive devices	X	-	X
Education inaccessible to persons with disabilities	X	X	X
Extra costs for accessing free health services	X	-	X
Inaccessible public buildings	X	X	X
Inaccessible transport system	X	X	X
Lack of awareness on the needs for persons with disabilities among decision-makers	X	-	X
Lack of decent accommodation	X	-	-
Lack of rights awareness among persons with disabilities	X	X	X
Negative attitudes of health workers	X	-	-
Persons with disabilities are self-limiting	-	X	X
Restricted participation in livelihoods	X	X	X
Social barriers	X	-	X

*Source*: Chichaya, T.F., Joubert, R.W. & McColl, M.A., 2017b, *Voices on disability in Namibia: Evidence for entrenching occupational justice in disability policy formulation*, Manuscript submitted for publication

Evidence on perspectives of disability policy stakeholders and their lived experiences is a prerequisite when conducting policy analysis. In addition to barriers experienced by persons with disabilities in Namibia, the participants suggested policy reforms. Table 5 presents the policy decisions suggested by each participant group.

**TABLE 5:** Recommended policy decisions to promote occupational participation among persons with disabilities.

Recommended policy decisions or direction to promote occupational participation with justice among persons with disabilities	Persons with disabilities	Disability policy decision-makers	Occupational therapists
Accessible communication format for all types of disabilities	X	X	X
Accessible municipal buses and local airplanes	X	-	-
Availing adequate budget for the policy provisions	-	-	X
Awareness raising to decision-makers and general population	X	-	X
Awareness raising to persons with disabilities	X	X	X
Consultation with all stakeholders in policy formulation	X	-	X
Employment creation by government	X	-	X
Ensuring accessibility in all schools	X	X	X
Ensuring equality at all times among persons with disabilities and those without disabilities	X	-	X
Establishing and enforcing accessibility standards to all buildings	-	X	X
Establishing rehabilitation centres	-	X	-
Establishing social enterprises	-	-	X
Financial support for SMEs for persons with disabilities	X	-	-
Legislation to support employment of persons with disabilities by companies	-	X	X
New transport system for persons with disabilities	-	X	-
Policy monitoring and evaluation mechanism	-	-	X
Vocational skills training	X	X	X

*Source*: Chichaya, T.F., Joubert, R.W. & McColl, M.A., 2017b, *Voices on disability in Namibia: Evidence for entrenching occupational justice in disability policy formulation*, Manuscript submitted for publication

Only 4 out of 17 recommendations for policy improvements were consistently mentioned by the three participant groups, namely, accessible communication formats for all disability types; awareness raising on disability issues; access to education; and vocational skills training. Disability policy decision-makers had the least number of suggested policy improvements. The occupational therapists who participated in the study provided perspectives for creating an occupationally just environment that promotes occupational participation among persons with disabilities in Namibia.

## Findings

### Constructing possible policy alternatives

As suggested by Bardach (2012), Step 3 is the construction of policy alternatives. The first alternative to be considered is to maintain the current state of things. The generated policy alternatives, which are based on research of suggested policy options from literature and the respective responses of persons with disabilities, occupational therapists and disability policy decision-makers in Namibia, fall into three main categories: access policy; support policy; and universal coverage policy.

### Maintaining the current state

If the current trends were to be maintained, the lives of persons with disabilities are not expected to improve. Firstly, the occupational injustice embedded in the disability policy and the policy environment in Namibia will remain; thus, the frustrations among persons with disabilities will continue to grow. Secondly, a laissez-faire approach is not likely to bring improvements to the lives of persons with disabilities because historically they have been oppressed and denied equal opportunities compared to the general population. The Government of Namibia has signed the UNCRPD, a reflection of commitment to improve the quality of life for persons with disabilities; thus, allowing the present conditions to prevail is equivalent to abandoning the commitment already made to the international treaty. Hence, this alternative was not pursued further in this study.

#### Access policy

To a greater extent the factors that restrict or limit persons with disabilities from participating in occupations of their choice are environmental. The access policy alternative seeks to address the environmental aspects that restrict persons with disabilities from participating in occupations in order to create accessibility to architectural infrastructure, information and services such as transport, education, employment, health and decent accommodation. In terms of the percentage of children in school in 2011, 69.2% of children with disabilities were enrolled, compared to 83.4% of children with no disabilities; 15.2% of children with disabilities had never attended school, compared with 4.7% among children with no disabilities (GRN 2012). In 2012 there was a roughly 90.0% unemployment rate among persons with disabilities compared to 27.4% for the general Namibian population (GIZ 2013). While Namibia reported better access to health services for persons with disabilities than other services, the significant barrier to accessing health services was lack of transport and long travelling distances (Eide et al. 2015). Thus, a policy alternative that improves access to the mentioned services has merit for further analysis.

#### Support policy

The support policy alternative focuses on the notion that persons with disabilities are disadvantaged and disability programmes are under-resourced; therefore more resources should be given to such programmes. Supporters of this policy alternative argue that it is a proactive approach to address disadvantages and barriers uniquely faced by persons with disabilities in order to achieve equality with the rest of the population without disabilities (Global Rights 2005). Currently Namibia is one of the few countries that provides a cash-based disability grant or pension and provides free healthcare services specifically to persons with disabilities in state facilities. Critics of the support policy alternative indicate that it fosters dependency on government and donor organisations, leaving persons with disabilities being construed as charity or welfare recipients with no capacity for contributions to socio-economic productivity of the country (Isaacs 2005). In addition, this policy option may be considered to perpetuate societal attitudes that persons with disabilities are a special group that require special treatment and charity; this defeats the goal of inclusion (Sheldon 2010). This policy alternative has merit to be considered for further analysis on its probable outcomes and trade-offs.

#### Universal coverage policy

From a universal coverage policy perspective, disability is viewed as one of many variables of the population; the structure of society should thus be targeted to provide for universal coverage of all members of the society. This policy alternative seeks to ensure that all persons with disabilities are catered for in the general community development policies. If this policy alternative is fully pursued, then disability may eventually cease to be a policy category (Bickenbach 2014). Those in support of universal approaches highlight that this is the only forward-thinking approach that allows for ensuring that services, products and environments are accessible and inclusive to the broadest population including persons with disabilities from the onset, thus eliminating the need for later modifications or adjustments to accommodate persons with disabilities or disability-targeted programmes (Story 2001).

Critics of the outset use of the universal coverage policy approach describe it as effective in a ‘utopian’ society; it does not address widespread imbalances and inequalities that are already being faced by persons with disabilities, which require corrective measures – hence the need for disability-targeted approaches as a means to achieve equality until such time as an ideal society is achieved (Sheldon 2010). Challenges still exist on how governments can target persons with disabilities but still abide by the principles of ‘universalism’. Disability is a complex phenomenon; thus, despite the presence of the Universal Declaration of Human Rights, there was need for the development of the UNCRPD. In addition affirmative action approaches remain relevant to target groups of persons who were formerly disadvantaged or marginalised to ensure their inclusion and equity. This policy alternative is worthy of further analysis.

### Setting evaluative criteria

Evaluative criteria are mental standards for evaluating the probable outcomes of identified policy alternatives; this is the fourth step of the eightfold path to policy analysis (Bardach 2012). The evaluative criteria that were set for this study were based on evidence obtained from the following data sources: disability policy documents; persons with disabilities; occupational therapists; and disability policy decision-makers in Namibia. Furthermore, the experiences and understanding of the authors of the disability policy environment and local context, as well as the ethos of the study, influenced the evaluative criteria that were selected. The latter were: *justice*; *inclusion*; *affordability*; and *political acceptability*. Explicitly providing these criteria allows readers to have a clear understanding of the reasons behind suggested policy options and recommendations.

Justice was selected as the first criterion in this study because it is about fairness and thus linked to rights of persons with disabilities as equal citizens. Rights-based criteria are favourable in selecting ‘better’ policies (Bardach 2012). In addition, justice falls within the domain of critical theory whereby the targeted outcomes include emancipating people from situations that suppress their rights. Thirdly, justice, as a criterion, is compatible with the human rights disability model used in this study and the UNRCPD principles. Lastly, the concept of justice is embedded in the occupational justice framework – the conceptual framework for this study. Thus, outcomes of policy alternatives were judged on how fair and unbiased they were towards permitting or promoting persons with disability to participate in occupations of their need and choice.

The second criterion is inclusion, which means determining whether implementation of identified disability policy alternatives results in persons with disabilities being accommodated in all spheres of society with clear involvement and participation in decision-making about their lives. This criterion satisfies the motto: ‘Nothing about us without us’. Furthermore, it encompasses the consideration of contextual factors that predispose people to exclusion as outlined in the occupational justice framework such as gender, disability or ethnicity.

The third criterion, affordability, was used to judge the resultant disability policy outcomes when suggested policy alternatives were assessed on the first two criteria of justice and inclusion. This means that an alternative with an outcome that is just and inclusive was further assessed in terms of the cost of resources required. Affordability depends on the type of economy, which constitutes the underlying occupational determinants of the occupational justice framework. Income status or wealth is also among the contextual factors that can contribute to outcomes of either occupational justice or injustice. Policy alternatives that are unaffordable will not be executed even if they meet the first two criteria.

Political action is a prerequisite for the implementation of any disability policy alternatives. Therefore, political acceptability was the fourth criterion used to judge the projected outcomes of policy alternatives after satisfying the previously mentioned three criteria. Political acceptability focused on assessing the political will for the implementation of policy options with least resistance.

### Outcomes matrix

Table 6 presents a tabulated outcomes matrix grid to project the probable outcomes of each policy alternative when subjected to the set evaluative criteria. This is Step 5 of the Bardach approach. The matrix shows the suggested policy alternatives in the rows and evaluative criteria in the columns (Bardach 2012; Kanna 2006). The projected outcomes for each policy alternative (access, support and universal coverage) were measured against the extent to which they satisfied the four evaluative criteria (justice, inclusion, affordability and political acceptability). All the selected evaluative criteria were given equal weighting; this was informed by the literature on the basis that if any outcome of a policy alternative would not satisfy any of the evaluative criteria, that policy option would not be useful. Thus, the higher the score, the more it meets the evaluative criteria; the policy option would therefore be most likely to address the identified needs of persons with disabilities. Conversely, the lower the score, the less it addresses their needs. A common qualitative interval measurement scale was used for assessing evaluative criteria as shown below:

- 3 = satisfies criteria: This means that the projected outcome of the alternative meets criteria to a substantial extent.
- 2 = moderately satisfies criteria: This means that the projected outcome of the alternative meets the criteria to a modest extent.
- 1 = minimally satisfies criteria: This means that the projected outcome of the alternative meets the criteria only to a negligible extent.
- 0 = does not satisfy criteria: This means that the projected outcome of the alternative completely fails to satisfy the criteria.

**TABLE 6:** Outcomes matrix illustrating policy alternatives and trade-offs for each evaluative criterion.

Policy alternatives	Evaluative criteria				Final scores
	1. Justice	2. Inclusion	3. Affordability	4. Political acceptability	
Access policy	3	3	2	2	10/12
Support policy	1	1	2	1	5/12
Universal coverage policy	3	3	1	1	8/12

*Source*: Adapted from Bardach, E., 2012, *A practical guide for policy analysis: The eightfold path to more effective problem solving*, 4th edn., Chatham House Publishers, Seven Bridges Press, New Yorkand and Kanna, B., 2006, ‘Access to Highly Active Anti-Retroviral Therapy (HAART) For HIV Infection In India’, *The Internet Journal of Law, Healthcare and Ethics* 4(2), 1–8

The higher the total score, the fewer trade-offs for the particular policy alternative, hence the more favourable it is. Table 6 shows that the policy alternative for access policy had the fewest trade-offs (10/12); the alternative of support policy had the most trade-offs (5/12). Following is an account of the probable outcomes of each policy alternative, and the trade-offs, based on how each alternative fared on evaluative criteria.

## Discussion

### Probable outcomes and trade-offs

Considering the trade-offs of probable outcomes if policy alternatives were to be implemented is Step 6 of the Bardach approach. The following discussion presents the probable outcomes for each disability policy alternative and the trade-offs when judged based on each evaluative criterion.

#### Alternative 1: Access policy – Outcomes and trade-offs

Seven out of the 12 barriers to occupational participation identified by stakeholders have to do with access, which places it high in priority. Similarly, the suggested policy interventions were based on improving access of persons with disabilities to transport; information; health services; education and training; employment; livelihood activities; and leisure activities. Thus, policy interventions that directly address access are relevant in addressing the needs of persons with disabilities in Namibia.

**Justice:** Access policy directly addresses the need for fairness in enabling persons with disabilities in Namibia to participate in occupations that are meaningful to them on an equal basis with other citizens. This policy alternative therefore satisfies the justice criterion.

**Inclusion:** Implementation of access policy is expected to foster inclusion because, by improving access to services and socio-economic spheres, persons with disabilities are able to participate in occupations that they choose and find meaning in. Furthermore, universal designs can be addressed under the access policy enhancing inclusion. This policy alternative satisfies the inclusion criterion.

**Affordability:** Considering the current economic challenges, and the budget cuts instituted by the Namibian government, implementation of this alternative will carry a financial implication. However, the establishment of the Department of Disability Affairs in the Office of the Vice President with its own budget provides for annual budgeting. Additionally, funding for implementing access policy can be a shared responsibility among other line ministries such as ministries responsible for education, transport, housing and labour. Therefore this alternative moderately satisfies the affordability criterion.

**Political acceptability:** The establishment of the Department of Disability Affairs in the Office of the Vice President in 2015 indicates a commitment to specifically addressing the needs of persons with disabilities in Namibia. The department has initiated plans for disability mainstreaming in other sectors; therefore access policy is most likely to have some acceptance and support from politicians. This alternative is considered to moderately satisfy the criterion of political acceptability.

#### Alternative 2: Support policy – Outcomes and trade-offs

The Government of Namibia has instituted some initiatives that are in line with support policy (e.g. disability grant and free health services for persons with disabilities). In 2015 the coverage of the disability grant was 65.0%, up from 24.0% in 2012 (GRN 2016). These support interventions specifically target persons with disabilities.

**Justice:** A policy that is entirely focused on a support policy perspective. This policy alternative can be considered to redress inequality by providing resources specifically for persons with disabilities to some extent; however, this is not primarily based on justice but on welfare. Therefore this alternative minimally satisfies the justice criterion.

**Inclusion:** This policy alternative does not favour inclusion because it portrays persons with disabilities as a special population that requires special treatment and charity. Persons with disabilities do not want to be perceived as those who are recipients of welfare assistance. They want to be perceived as equal, economically productive citizens. This alternative minimally satisfies the inclusion criterion.

**Affordability:** This policy alternative will demand more financial resources in the form of providing more free services for persons with disabilities. Payment of such provisions will be mainly derived from taxpayers. The Government of Namibia has increased the flat-rate grant of NAD 100.00 (about US $8.30) to make it NAD 1200.00 (about $100.00) per month for the financial year 2017 and 2018 (GRN 2017b). This percentage increase is an indication that government is not focused on huge spending on welfare. In general the 2017 and 2018 budget for the social sector has been increased while other sectors have experienced budget cuts in line with the Harambee Prosperity Plan, which emphasises that no one must be left behind (GRN 2016; 2017b). Therefore this alternative moderately meets the affordability criterion.

**Political acceptability:** Adopting a support policy as an alternative for addressing the needs of persons with disabilities may not have much political acceptability because of the perceived risk of dependency. Furthermore, taxpayers will not be expected to be very supportive of this policy alternative as it will be funded by them. Previously there were reports of mismanagement of resources by organisations for persons with disabilities, which led to closure of their offices; the donors and government declined to provide further support to such organisations (Thihenuna 2015). This implies the need for reviewing the system of policy implementation. Furthermore, the government cited lack of sustainability and creating dependency and thus did not accept a 2004 civic group proposal for the introduction of a cash-based basic income grant, which was to be given to all Namibians unconditionally (Melber 2016). This policy alternative minimally satisfies the political acceptability criterion.

#### Alternative 3: Universal coverage policy – Outcomes and trade-offs

This policy alternative seeks to encompass the broadest diversity of the population in which persons with disabilities are considered as part of a diverse population. This eliminates the need for a disability-specific policy. However, since political independence, Namibia has focused on redressing the results of the apartheid regime such that minority groups, and previously disadvantaged ethnic groups, are given preference. This is opposite to the universal coverage alternative.

**Justice:** The universal coverage disability policy option is based on the rights of every person and the creation of a just society for a diverse population. Therefore it fully satisfies the justice criterion.

**Inclusion:** The universal coverage policy alternative ensures that persons from diverse backgrounds, including persons with disabilities, are equally included in society and therefore satisfies the criterion of inclusion.

**Affordability:** Significant resources will be required for the implementation of this policy alternative, cutting across the socio-economic sectors, addressing the needs of diverse populations of all age groups from rural and urban areas. Despite Namibia being graded as an upper middle income earning country, the current economic challenges are considered to translate into this alternative minimally satisfying the criterion of affordability.

**Political acceptability:** While the universal coverage approach has ideal outcomes, its full implementation will not receive much support from the government. Firstly, persons with disabilities have been historically disadvantaged and a redress is required. Secondly, the recent establishment of the Department of Disability Affairs to address the concerns of persons with disabilities will cease to be relevant if a universal approach is adopted; thus, government is not likely to make a huge leap in policy change. It is reasonable to expect the current Namibian government not to fully accept this policy alternative; thus, it minimally satisfies the criterion of political acceptability.

### Discussion on Steps 7 and 8 of the Bardach approach

Steps 7 and 8 of the Bardach approach entail deciding on and telling the story, respectively. Detailed application of these two steps is beyond the scope of this study. Firstly, this is because the study focused on generating evidence that can be used for disability policy formulation in Namibia by presenting different policy alternatives and their trade-offs without being prescriptive on a single disability policy alternative (deciding). Secondly, telling the story (Step 8) involves developing a communications plan, going back to the field to engage different stakeholders with the results and seeking their buy-in on a selected disability policy alternative.

### Summary of discussion for Steps 1–6

Steps 1 and 2 (defining the problem and gathering evidence) were addressed in Phases 1 and 2. In Step 3, the following three disability policy alternatives were generated: access policy, support policy and universal coverage policy. Four evaluative criteria were set in Step 4: justice, inclusion, affordability and political acceptability. For Step 5 an outcomes matrix was presented to project the probable outcomes when each policy alternative is judged based on the set evaluative criteria. In Step 6 a discussion on the trade-offs for each disability policy alternative is provided concerning promotion of occupational justice using a critical disability theory lens. The access policy alternative had the fewest trade-offs – that is, the highest score – followed by the universal coverage policy alternative and lastly the support policy alternative.

The policy analysis has prompted naming a new form of occupational injustice as occupational inconsideration. Findings from the policy documents and participants in Phases 1 and 2 of the study reveal a disparity between perceptions of disability policymakers and persons with disabilities on the occupational needs of persons with disabilities. This disparity results in policymakers designing and approving disability policies that are inconsiderate of the need to ensure occupational participation among persons with disabilities.

The adopted dictionary definitions for *inconsiderate* include ‘without due regard for the rights or feelings of others’; ‘insensitive’; ‘ill-advised’. Occupational inconsideration thus exists when disability policymakers design policies or services targeting persons with disabilities without carefully considering how occupational participation for the intended target audience will be achieved. Further research of this concept, including involvement of a broader range of stakeholders in disability and rehabilitation services, is necessary.

## Conclusion

The purpose of the study was to develop disability policy alternatives and present their probable outcomes and trade-offs based on the occupational justice framework and the eightfold path to policy analysis. Three disability policy alternatives emerged from the evidence in this study: access policy; support policy; and universal coverage policy. The access policy is more likely to result in achievement of fairness and increased occupational participation among persons with disabilities in Namibia in the present context. These findings highlight the relevance of introducing occupational justice into disability policy formulation.
